# Characterization of post-transfusion anti-FEA 1 alloantibodies in transfusion-naive FEA 1-negative cats

**DOI:** 10.1177/1098612X221094502

**Published:** 2022-05-05

**Authors:** Alyssa Cannavino, Dana LeVine, Marie-Claude Blais

**Affiliations:** 1 Department of Clinical Sciences, Faculty of Veterinary Medicine, Université de Montréal, Saint-Hyacinthe, QC, Canada; 2 Department of Clinical Sciences, Bailey Small Animal Teaching Hospital, College of Veterinary Medicine, Auburn University, Auburn, AL, USA

**Keywords:** Feline erythrocyte antigen, alloantibodies, transfusion, crossmatch, *Mik* antigen

## Abstract

**Objectives:**

The aim of this study was to characterize anti-feline erythrocyte antigen (FEA) 1 alloantibodies following sensitization of FEA 1-negative cats, including their rate of appearance, agglutination titer over time and immunoglobulin class. A secondary aim was to obtain polyclonal anti-FEA 1 alloantibodies to increase the availability of FEA 1 blood typing. We also describe a case study documenting an acute hemolytic transfusion reaction in a transfusion-naive FEA 1-negative feline patient that received FEA 1-positive blood.

**Methods:**

In this prospective clinical study, 35 cats with blood group type A underwent extensive blood typing for FEA 1–5. Two cats were identified as FEA 1-negative; these cats were transfused uneventfully with 50 ml of FEA 1-positive, but otherwise compatible, packed red blood cells. Post-transfusion blood samples were collected routinely as long as anti-FEA 1 alloantibodies were detected. Appearance of anti-FEA 1 alloantibodies was detected using a gel column crossmatch method.

**Results:**

Anti-FEA 1 alloantibodies were detected as early as 5 days post-transfusion and remained detectable for over 400 days in one cat. Agglutination titers in both cats were relatively weak (1:1 to 1:8). The main immunoglobulin class was IgM.

**Conclusions and relevance:**

Transfusion of FEA 1-negative, transfusion-naive cats with FEA 1-positive blood results in production of post-transfusion anti-FEA 1 alloantibodies as early as 5 days post-transfusion. Our results confirm the potential immunogenicity of FEA 1 and support crossmatching prior to a blood transfusion, even in transfusion-naive cats. Further studies are needed to better document the clinical importance of these post-transfusion antibodies, as well as to facilitate routine blood typing for the FEA 1 antigen in cats.

## Introduction

Over the past decade, our knowledge of feline transfusion medicine has grown significantly, and as a result, the clinician’s approach to transfusing cats is continuously evolving. It is standard practice to type cats within the AB system prior to a blood transfusion due to the presence of naturally occurring alloantibodies (NOAbs) and the potential risk of severe acute hemolytic transfusion reactions. AB typing is an essential part of feline transfusion; however, while current recommendations are to crossmatch a cat prior to a first transfusion,^1^ the benefits of this have been debated in some recent papers.^2,3^ However, the existence of feline erythrocyte antigens (FEAs) outside the AB system has led to questions about their potential clinical impact and the limitations of our current understanding of feline transfusion medicine. Most notably, the discovery of the *Mik* antigen in 2007 gave way to further research on non-AB group-related FEAs and their associated alloantibodies, and the role they may play in feline blood transfusion reactions. Previous studies evaluating NOAbs in transfusion-naive cats report a prevalence of 0–29%.^4–9^ Further characterization of these FEAs was unavailable until a recently published study by Binvel et al^10^ documented and characterized five new FEAs, named FEA 1–5, by evaluating crossmatch incompatibilities based on the presence of NOAbs. In the study by Binvel et al,^10^ only FEA 1-negative status was associated with a higher risk of having NOAbs. It is unknown whether one of these newly discovered FEAs may correspond to the lost *Mik* antigen, for which testing is no longer available.^6,10^ This highlights the importance of banking anti-FEA alloantibodies to allow for continued research on the subject. Information regarding the antigenicity of these NOAbs and the role they might play in transfusion medicine practice is currently lacking. Furthermore, it is unknown whether cats that test negative for a given antigen and that lack NOAbs against this antigen would develop clinically significant antibodies following a blood transfusion.

The primary aim of this study was to characterize the development of anti-FEA 1 alloantibodies in FEA 1-negative, transfusion-naive cats lacking NOAbs, including their rate of appearance, agglutination titer over time and immunoglobulin class. A secondary aim was to bank polyclonal anti-FEA 1 alloantibodies over time in order to increase their availability and to facilitate future FEA 1 research and clinical blood typing. We also describe a case study documenting an acute hemolytic transfusion reaction in a transfusion-naive FEA 1-negative feline patient that received FEA 1-positive blood.

## Materials and methods

### Animals

All type A cats from the Université de Montréal’s Centre Hospitalier Universitaire Vétérinaire (CHUV) feline blood bank (n = 11) and the CHUV feline teaching colony (n = 24) were prospectively typed for FEA 1–5 using previously obtained and banked polyclonal feline alloantibodies from a prior study performed at our facility.^10^ These cats were also evaluated for the presence of NOAbs using a gel column technique (ID-MTS Micro Typing system; Ortho-Clinical Diagnostics), as described in the study by Binvel et al^10^ and detailed below. A complete physical examination, full bloodwork (hematology, chemistry and total thyroxine level) and an echocardiogram by a board-certified cardiologist were performed on FEA 1-negative cats to assess their eligibility as potential blood transfusion recipients. A donor cat from the CHUV’s feline blood bank was selected for each recipient based on their clinical availability and FEA 2–5 compatibility with the recipient, meaning they had to be FEA 1-positive but otherwise have a similar blood type profile to the recipient cat to optimize selective sensitization against FEA 1. A major crossmatch was performed between the donor and recipient cat, as described below, to confirm their compatibility prior to transfusion. The study was approved by the Institutional Animal Care and Use Committee of the University of Montreal, which adheres to the ethical requirements of the Canadian Council on Animal Care.

### Technique

Crossmatching and blood typing were performed using the gel column technique. Briefly, following centrifugation of EDTA blood, 5 µl of red blood cell (RBC) concentrate was added to 0.5 ml of an antibody enhancement solution (low ionic strength saline; Ortho-Clinical Diagnostics) to obtain a 0.8% RBC suspension. A total of 50 µl of the RBC suspension and 25 µl of the anti-FEA 1 polyclonal antiserum (banked serum from a known FEA 1-negative cat with NOAbs)^10^ was placed on top of a gel column on a buffered saline ID card (ID-MTS Micro Typing system; Ortho-Clinical Diagnostics). The card was incubated at 37°C for 15 mins (ID incubator 37 SI I; DiaMed Microtyping System) and then centrifuged for 10 mins (ID centrifuge 12 S II; DiaMed Microtyping System). Results were interpreted according to a standardized classification and graded from 0 (negative) to 4+ (strongly positive). The RBCs’ migration through the gel was scored according to the following scale: 4+ = all RBCs were agglutinated and formed a red line on the top of the gel; 3+ = most RBCs were agglutinated on the top half of the gel with some retained on the surface of the gel; 2+ = RBC agglutinates were predominantly observed in the lower half or were dispersed throughout the gel; 1+ = few RBC agglutinates were dispersed in the lower half of gel, with most of the RBCs found at the bottom of the gel; and 0 = all RBCs were at the bottom of the tube (none agglutinated).

### Sensitization of FEA 1-negative cats

The day before sensitization, blood was collected from the selected blood donor using the CHUV’s standardized feline blood donor program procedure. After sedation with the CHUV’s blood donor program’s standard protocol (intravascular ketamine and diazepam), a total of approximately 50 ml of whole blood was aseptically collected from the jugular vein of the donor cat and stored at 4°C until use. The blood was allowed to passively separate via gravity overnight, and, on the day of transfusion, the plasma was removed and only the RBCs were used for transfusion. This was carried out to reduce the volume of transfused blood and minimize any potential adverse transfusion reactions.

To avoid fluid overload, on day 0, approximately 5 ml/kg of blood (ie, the same volume to be transfused) was collected from each recipient cat under sedation prior to transfusion. For a first-time transfusion, the recipient cat received a transfusion of 5 ml/kg of packed RBCs from the selected donor, over a maximum of 4 h, to reflect a standard blood transfusion administration. For any subsequent transfusions, the recipient cat received a total of 5 ml of packed RBCs over a maximum of 1 h. Additional transfusions were administered only once the FEA 1 agglutination titer was no longer detectable (ie, an agglutination titer of 0). This was carried out to maximize data acquisition due to the low number of FEA 1-negative cats available for this study, and it allowed for the evaluation of repeat seroconversion in cats with anti-FEA 1 alloantibodies that had fallen under the threshold of detection. In addition, the goal was to maximize storage of anti-FEA 1 alloantibodies with repeat seroconversion when possible. The recipient cats were closely monitored throughout the transfusion as per standard practice, in accordance with recent Association of Veterinary Hematology and Transfusion Medicine (AVHTM) guidelines,^1^ and every 4 h for the following 24 h (heart rate, respiratory rate, pulses, mucous membranes, mental state, blood pressure, temperature and urine color [ie, hematuria or hemoglobinuria]). Packed cell volume (PCV), total solids and serum color were evaluated 2 h post transfusion. Total bilirubin levels were performed 3–5 days post transfusion.

### Rate of appearance of alloantibodies and agglutination titers

The aforementioned gel column technique was used to detect the development of FEA 1 alloantibodies in either the serum or plasma of the recipients. Testing was performed daily for the first week, once a week for the first month and then once a month as long as alloantibodies were detected. For each evaluation, an auto-control was performed using RBCs from the sensitized cat itself. In addition, RBCs from two known FEA 1-positive cats and an FEA 1-negative cat were used as positive and negative controls, respectively. Anti-FEA 1 alloantibodies were considered present when the RBCs were trapped on top of or within the gel column containing FEA 1-positive RBCs (corresponding to an agglutination grade of ⩾1+), whereas the reaction was considered negative if the RBCs passed through the gel and formed a pellet at the bottom (corresponding to an agglutination grade of 0). The agglutination titer was defined as the highest serum dilution where a positive agglutination reaction was observed and was expressed as the whole number corresponding to the serum dilution.

### Immunoglobulin class

To determine the immunoglobulin class, agglutination titer was determined following treatments with sulfhydryl reagents (dithiothreitol [DTT] and 2-mercaptoethanol [2-ME]), which break the disulfide bonds of IgM and abolish their agglutinating and complement-binding activities. Frozen serum from both cats at peak post-transfusion antibody titers was used (21 days after the second transfusion for the first index cat, and 14 days post-transfusion for the second index cat). Serum was incubated in a 1:1 ratio with either phosphate-buffered saline, DTT 0.01 M or 2-ME 0.1 M.^11^ The suspensions were incubated at 37°C for 60 mins and then the agglutination titer was determined as described above.

## Results

A total of 35 cats were typed for FEA 1 (11 from the CHUV’s feline blood bank and 24 from the CHUV’s feline teaching colony); of these, three were typed FEA 1-negative but only two of these did not have any NOAbs and were thus selected as recipient cats (index cat 1 and 2). Both cats were male neutered domestic shorthair cats aged approximately 3 years, and were both healthy based on physical examination, full bloodwork and echocardiography.

Index cat 1 received a total of four transfusions: one full-volume transfusion (5 ml/kg) and three subsequent transfusions of 5 ml packed RBCs administered once anti-FEA 1 alloantibodies were no longer detected. The number of days lapsed between the first and second, second and third and third and fourth transfusions were 71, 163 and 105 days, respectively. Transfusions 1 and 2 involved blood from the same donor (donor 1) and transfusions 3 and 4 were from a second donor (donor 2). Index cat 2 received a single full-volume transfusion with blood from donor 2. All blood transfusions were uneventful, with no clinical signs of acute or delayed transfusion reactions observed. Clinically, the color of the cats’ mucous membrane and vital parameters remained stable. No evidence of intravascular hemolysis was noted on serum or urine evaluation. Laboratory results were all within normal range, including post-transfusion bilirubin levels. All recipient auto-controls remained negative for the entire duration of the study.

**Figure 1:**
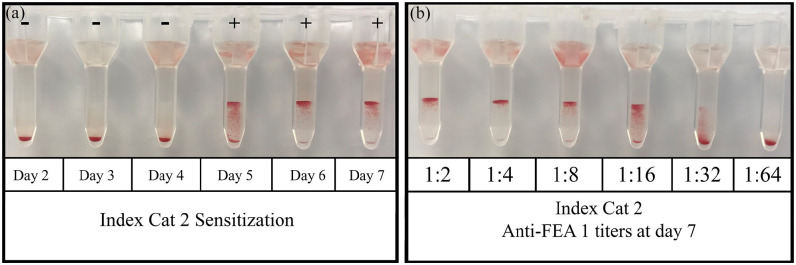
(a) Crossmatches between feline erythrocyte antigen (FEA) 1-negative index cat 2 and its respective FEA 1-positive donor at days 2–7 post-transfusion. Index cat 2 developed anti-FEA 1 alloantibodies as early as day 5 post-transfusion. (b) Index cat 2’s anti-FEA 1 alloantibody agglutination titers at day 7 post-transfusion reaching a maximum of 1:32. Crossmatching was performed between index cat 2 and its respective FEA 1+ donor

**Figure 2:**
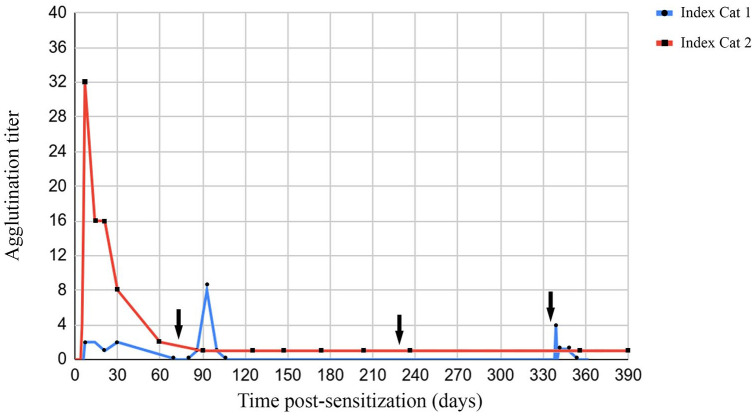
Graph indicating the anti-feline erythrocyte antigen (FEA) 1 agglutination titers over time of index cats 1 and 2. Day 0 corresponds to the day of primotransfusion for both index cats 1 and 2. The arrows correspond to when index cat 1 received its second, third and fourth transfusions, administered 72, 232 and 334 days after initial transfusion, respectively

A positive anti-FEA 1 agglutination reaction was first observed on day 7 for index cat 1 (titer of 1:2). In index cat 1, anti-FEA 1 alloantibodies were no longer detected 60 days following its first transfusion. The alloantibody titer reached a maximum of 1:8 at day 21 following its second transfusion, but alloantibodies were not detectable at 60 days post-transfusion. No anti-FEA alloantibodies were identified following the third transfusion. For index cat 1’s third transfusion, owing to a technical problem with the blood filter, there was some volume loss, and it is estimated that the cat may have received 3 ml of RBCs rather than 5 ml. For the fourth transfusion, antibodies were first detected at day 5 and this was also when alloantibody titers were highest (1:4). By day 14, antibodies were no longer detectable. For index cat 2, a positive anti-FEA agglutination reaction was first observed on day 5 and maximal titers were noted on day 7 (1:32) (Figure 1a,b). Alloantibody titers rapidly declined over time but remained persistently weakly positive (1:1) 421 days after transfusion (at the time of writing) (Figure 2). Maximal agglutination titers were associated with a 3+ grade. Antibody titer was found to be more sensitive than agglutination grade. Auto-controls for both index cats remained negative for the entirety of the study. All incompatible (positive) reactions were repeatable when using two positive controls and of comparable agglutination strength, and all compatible (negative) reactions were negative when using the negative control, thus increasing the likelihood of specificity of anti-FEA 1 alloantibodies being identified. Additionally, positive controls were selected based on compatible FEA 2–5 typing profiles with the recipient cats to further maximize specificity.

For immunoglobulin testing, a serum sample with peak agglutination titers for each cat was selected (day 21 after second transfusion for index cat 1 and day 14 for index cat 2). Agglutination titers fell to zero following treatment with either DTT or 2-ME, implying that the causative immunoglobulins were cleaved by the sulfhydryl reagents and therefore anti-FEA 1 alloantibodies were primarily of IgM class.

### Case study

An 18-year-old female spayed domestic shorthair cat was presented to Iowa State University Teaching Hospital (ISU) for progressive azotemia in the context of previously diagnosed stage IV proteinuric, hypertensive chronic kidney disease (CKD). The day following admission and initiation of fluid therapy, the cat’s PCV was 14%. The anemia was characterized as normocytic, normochromic and non-regenerative. Blood typing using an immunochromatographic strip test (CHROM Method, Lab Test A + B; Alvedia) confirmed the cat was type A, and it was transfused with 27 ml of non-crossmatched type A whole blood administered over 3 h. Prior to transfusion, the cat’s serum and urine were clear. Urinalysis showed isosthenuria and mild glucosuria, and urine culture was negative. During and after the transfusion, the cat urinated blood-tinged urine. One hour post-transfusion, PCV was 17% and serum was hemolyzed. The following day, PCV had dropped back down to 14% and serum was still hemolyzed and remained so for 5 days afterwards. Blood typing was repeated after the hemolytic transfusion reaction and confirmed the cat was indeed type A. The day following the initial transfusion, the cat was crossmatched with all available feline donors at ISU and was incompatible with all donors. A transfusion of 40 ml of crossmatch-compatible DEA 1-negative canine blood was administered over 2.5 h without any immediate complications. Post-transfusion PCV was 29% but decreased to 14% 4 days later. The cat received a darbepoietin injection and was discharged from the hospital with supportive treatment.

**Figure 3:**
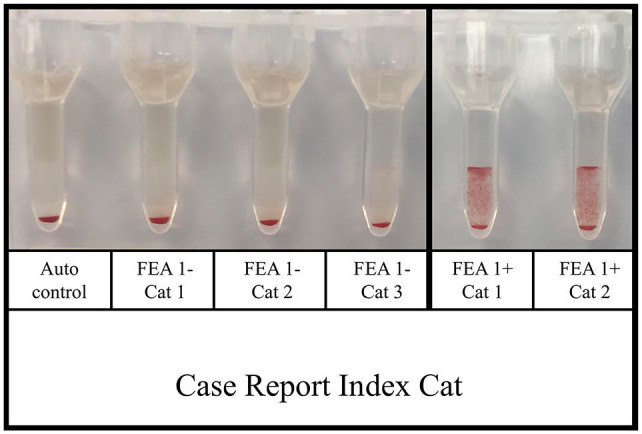
The case report index cat was crossmatch-compatible with three previously typed feline erythrocyte antigen (FEA) 1-negative cats and two FEA-1 positive cats. The index cat’s auto-control at the time of testing was negative

A pre-transfusion plasma sample from the index cat (taken at admission) was submitted to the University of Montreal’s CHUV for extensive blood typing. Using an RBC sample from 5 days post-transfusion (the only RBC sample available for study), the index cat was confirmed to be FEA 1-negative and positive for FEA 2–5. The index cat was crossmatched against three known FEA 1-negative cats that it was compatible with and two known FEA 1-positive cats that it was incompatible with (2+ incompatibility) (Figure 3). Pre-transfusion RBCs from the index cat were not available, but an auto-control using RBCs and plasma available from 5 days after initial transfusion was negative. This was performed to rule out auto-agglutination that might interfere with crossmatch interpretation. Pre-transfusion plasma titers, established by crossmatching against FEA 1-positive RBCs, were 1:256 (pre-transfusion) and 1:512 (5 days after initial transfusion). The index cat was re-presented to ISU for a re-evaluation of its CKD and anemia 9 days after the initial admission date and 4 days after hospital discharge. PCV was 19% and its general condition was stable. A 23 g unit of type A pRBCs from the CHUV’s only FEA 1-negative feline blood donor had been shipped overnight to ISU. This unit was crossmatch-compatible with the index cat and the blood was administered over 3 h. Post-transfusion PCV was 23%. The cat was hospitalized for 14 h after the transfusion was begun and vital parameters, including mental status, heart rate, blood pressure, pulse strength, mucous membranes, respiratory rate and urine (for presence of pigmenturia), were monitored closely during this period before the cat’s discharge. The cat then had a re-evaluation with its local veterinarian 5 days later, at which time it was clinically stable and had a PCV of 30%.

## Discussion

Our study demonstrated that FEA 1 appears to be immunogenic, with transfusion-naive, FEA 1-negative cats developing post-transfusion anti-FEA 1 alloantibodies when transfused with FEA 1-positive blood. Alloantibodies developed as early as 5 days post-transfusion, similar to a previous report of post-transfusion sensitization in transfusion-naive cats in which post-transfusion non-AB incompatibilities occurred as early as 2 days post-transfusion with a median of 5 days.^5^ In our study, FEA 1 sensitization resulted in low to moderate titers of post-transfusion anti-FEA 1 alloantibodies (maximum titer of 1:32). Interestingly, the antibody titers and duration were considerably different between the two cats in this study, with antibodies from index cat 1 lasting 14–30 days but >1 year for index cat 2. This may reflect inter-individual differences of the immune system response with regard to antibody production and the degree of reaction to different donors. It is possible that FEA 1 antigen expression and antigenicity vary between cats, and since two different donors were used in this study, differing antigenicity may have influenced the post-transfusion sensitization of both index cats. Another factor to consider is whether transfusion of increased blood volume (and therefore greater absolute quantity of RBCs) is proportionally related to antigenicity. If so, the subsequent lower-volume blood transfusions administered to index cat 1 may explain the lackluster alloantibody production following transfusions 2–4. It was decided to only administer 5 ml of RBCs for these subsequent transfusions out of concern for possible adverse reactions following re-transfusion, but it is unknown whether index cat 1’s sensitization would have differed had full-volume (5 ml/kg) transfusions been administered instead. There was some volume loss during index cat 1’s third transfusion due to a technical problem with the blood filter, though it cannot be confirmed whether this was related to the lack of antibody production following the third transfusion.

Also of consideration is whether the strength and clinical importance of transfusion-induced anti-FEA alloantibodies are comparable to those of NOAbs. In our study, post-transfusion antibody titers ranged from 1:1 to 1:32, whereas the cat described in the above case study that had an acute hemolytic transfusion reaction had a much higher NOAb titer of 1:256. When obtaining these titers, the same positive controls were used, and there was a comparable agglutination grade and titer between both controls. In the study by Binvel et al,^10^ NOAb titers ranged from 1:1 to 1:32 for all FEA, with a range of 1:4–1:8 for FEA 1, specifically. Titers obtained for anti-*Mik* alloantibodies ranged from 1:1 to 1:64.^4^ Direct comparison of titers from different studies is difficult, as different cats may express different RBC antigenicity. Nonetheless, it is interesting to note that the case report index cat had notably higher NOAb titers, similar to how type B cats typically have significantly higher anti-A titers that reach up to 1:2048 and can result in severe, acute hemolytic transfusion reactions (compared with anti-B titers in type A cats, which are typically <1:32 and associated with less severe, delayed hemolytic transfusion reactions).^12^

Transfusion-induced antibodies in this study were also principally composed of immunoglobulin type IgM, which is the main type of immunoglobulin in cats, especially with regard to anti-A NOAbs in type B cats. IgM antibodies have a pentamer form compared with IgG monomers, and therefore have a better agglutination capacity and can be more potent contributors to hemolytic transfusion reactions.^12^

The secondary aim of the study, which was to collect and bank serum containing anti-FEA 1 antibodies from sensitized cats, was also achieved. Though this is not a long-term solution, banking these serum samples remains essential for novel typing of cats for FEA 1 status and allows for continued studies on this subject. Monoclonal antibody production would allow for a more perennial supply of FEA 1–5 typing reagent.

This study has several limitations. The number of cats included in the study (n = 2) was small, owing to the low prevalence of FEA 1-negative cats without NOAbs at our facility. This prevalence (5.7% of the 35 cats screened) is lower than the estimated prevalence within the tested feline population at the CHUV in the study by Binvel et al^10^ (13.5% of the 258 cats tested). This is likely secondary to the small pool of cats available for study, despite the teaching colony being composed of a rather heterogeneous population of rescued cats that are strays or relinquished cats from various individuals and shelters in the Montérégie, Quebec area.

These two cats responded differently to their transfusions with regard to the extent and duration of their seroconversion, though both seroconverted and neither developed evidence of hemolysis. Lack of hemolysis is not surprising as they were transfused when they did not have any detectable anti-FEA 1 alloantibodies. Inter-individual variability is to be expected, though inclusion of more subjects would provide more information on the extent of this variability. The subjects in our study were young, healthy cats, and therefore not fully representative of the typical feline population requiring a blood transfusion. It is unknown to what extent age, sex and health/disease status may have influenced the results we obtained. Additionally, as mentioned previously, we arbitrarily decided to use a total volume of 5 ml of RBCs for any subsequent transfusions to limit potential adverse effects; however, this is not a representative volume of what is typically used in standard clinical practice and we cannot rule out the possibility that an increased volume of RBCs is correlated to increased antigenicity and thus, potentially, increased risk of post-transfusion antibody production.

The use of positive and negative controls strengthens the likelihood that produced anti-FEA 1 alloantibodies in this study were specific for FEA 1; however, we cannot entirely rule out the possibility of other interactions against as yet unidentified FEAs, though this is considered unlikely. Lastly, we performed crossmatches using the gel column technique. There is no standardized reference method for crossmatching in veterinary medicine, though most previous veterinary transfusion medicine studies used the tube agglutination method. The gel column method has been shown to have an excellent degree of concordance with the tube agglutination method.^4,6^

## Conclusions

Transfusion of FEA 1-negative, transfusion-naive cats with FEA 1-positive blood results in the production of post-transfusion anti-FEA 1 alloantibodies as early as 5 days post-transfusion. There appears to be inter-individual variability regarding time to development of antibodies, duration of antibodies and antibody titer over time. Our results confirm the potential immunogenicity of FEA 1, as some cats appear to have strong anti-FEA 1 NOAbs that can result in an acute hemolytic reaction secondary to receiving blood from a FEA 1-positive donor, as in the documented case report. These results support crossmatching prior to a blood transfusion even in transfusion-naive cats, to prevent sensitization of cats, which can complicate subsequent blood transfusions. Though hemolytic transfusion reactions due to non-AB incompatibilities appear to be rare, and systematic crossmatching does involve additional financial cost and time, we believe that prevention of unnecessary transfusion reactions (however rare) is in the best interest of the patient and conforms with current best practice transfusion medicine. Further studies are needed to better document the clinical importance of these post-transfusion antibodies, as well as to facilitate routine blood typing for the FEA 1 antigen in cats.
